# How the forensic multidisciplinary approach can solve a fatal dog pack attack

**DOI:** 10.1007/s12024-023-00746-8

**Published:** 2023-11-08

**Authors:** M. Di Nunzio, A. Della Valle, A. Serino, F. Corrado, C. Di Nunzio

**Affiliations:** 1 University of Barcelona, Barcelona, Spain; 2 Universityo of Pavia, Pavia, Italy; 3 Hospital of Caserta, Caserta, Italy; 4 Istituto Zooprofilattico Sperimentale del Mezzogiorno, Portici, Italy; 5 CEINGE, Napoli, Italy; 6 Università Degli Studi Di Napoli Federico II, Napoli, Italy

**Keywords:** Fatal dog attack, Dog bites, Dog territory defense, Dog DNA identification, Autopsy, Forensic science

## Abstract

The authors present the case of a 61-year-old man found dead in an agricultural plot. The first investigation of the scene revealed the corpse laid face up in a spot of partially dried blood, next to an olive tree. His face, arms, legs, and abdomen showed signs of severe contusion and laceration of dogs’ bite wounds. Next to the victim, an olives bin had been found overturned on the ground. A multi-disciplinary approach, including crime scene analysis, autopsy findings, veterinary animals review, odontologist bite mark study, and forensic genetics DNA correlations, was performed. The present case is a documented watchdogs lethal pack attack and provides an example of how to recognize the more active participants thanks to their odontological alterations. It could be considered the first described dog pack attack case solved by dysgnathia alteration. Comparisons between the dental casts obtained from the dogs and the inflicted wounds were made, resulting in positive correlations between the injuries and the dental arches from two of the six involved dogs, thanks to dental abnormalities and DNA founding. The victim’s clothes were also compared with the dogs’ dental casts, confirming that they were the most active participants during the pack attack. Dogs’ DNA was finally matched with saliva traces found on victim’s clothes and skin bite marks.

## Introduction

Dog attacks, specifically dog pack attacks, represent a fatal risk because of the severe injuries that can result in death of the victim [1, 2]. Non-fatal bites tend to be found, as in our case, on the lower limbs and face [3]. The concept of dog pack attacks was described for the first time in 1958 [4]; resulting injuries were described as a combination of biting, clawing, and crushing forces resulting in wounds with a characteristic pattern of punctures, lacerations, and avulsions of skin and soft tissues [1, 5–7]. These attacks are fortunately rare in our society, but when they happen, fatal results may occur. In Europe, deaths caused by dog attack have an incidence of 0.009 per 100,000 inhabitants, a little bit higher than Australia (0.004), but is comparable to estimates from the USA (0.011) and Canada (0.007) [8]. Individual implication grade analysis in a dog pack attack is extremely difficult to solve.

A careful forensic multidisciplinary investigation was conducted by authors, including a detailed analysis of the death scene, the victim’s body damages, and the animals suspected of the attack. This article presents the first described and solved fatal Cane Corso dog pack attack case, due to the dysgnathia conditions of some of the involved dogs.

## Case report

### Analysis of the death scene

**Fig. 1:**
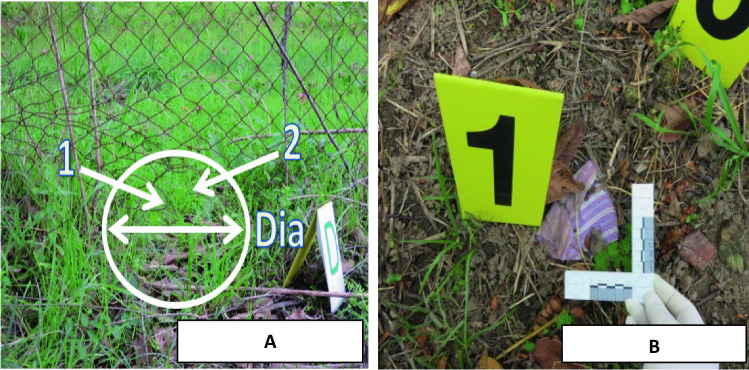
**A** View of the hole in the wire mesh, where the dogs presumably walked through. “Dia” indicate the diameter of the hole (60 cm). “1 and 2” indicated two mesh broken extremities where dark tufts were found. **B** Detail of a shirt fragment collected in the second crime scene inspection (samples matched to the victim’s shirt)

**Fig. 2:**
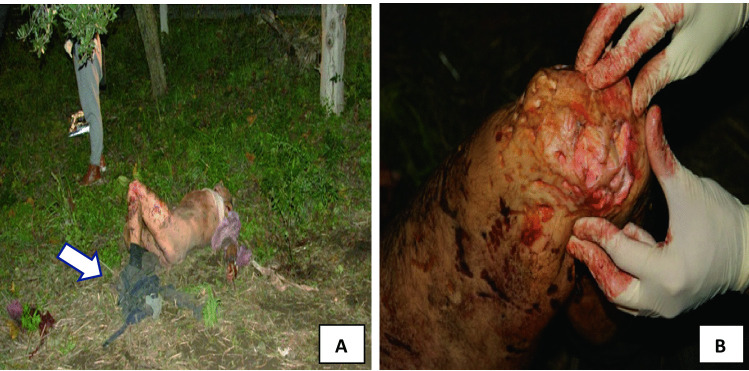
**A** Death scene. The corpse found close to the olive tree. Details of the trousers that had been found pulled down to the ankles.** B** Left knee extensive damage (the bite lesions were identified as postmortem due to no clinical evidence of tissue vitality)

A 61-year-old man was found dead in an agricultural plot. The victim was occasionally there to pick olives for the owner of the agricultural plot. When officers came to investigate the crime scene, a dark dog that had been guarding the victim’s body wandered away and disappeared through a hole in a wire mesh (Fig. 1A). The victim’s upper clothes were torn, and pieces of his sweater and shirt were found around him (Fig. 1B). His trousers had been found pulled down to the ankles (Fig. 2A), further indicating that he had been dragged. The victim’s first on-site examination revealed face and abdomen injuries, as well as severe injuries of arms and left knee (Fig. 2B). During the “on-the-spot” investigation, six black Cane Corso dogs were found in the area surrounding the death scene. Four dogs belonged to one owner, and they were tagged as Dog_1, Dog_2, Dog_3, and Dog_4. Another person, living near the crime scene area, owned the other two dogs (Dog_5 and Dog_6). In Dog_1’s mouth, a mixed saliva-blood substance was found and collected for future comparisons. All the six dogs were taken to the kennel for further investigation. Due to the complexity of the injuries and the number of seized dogs, the prosecutor asked to determine the dogs’ involvement in the killing of the victim.

### Autopsy findings and victim’s bite lesion analysis

A general examination of the undressed body revealed traumatic wounds caused by several and deep dog bites. It was found that the deepest indentations, present in the upper right limb at the brachialis level, had caused losses of muscle-cutaneous substance at depths ranging from 2.5 to 4 cm. The right brachial artery was found slashed, suggesting heavy and massive blood loss, which could be the main cause of the victim’s death.

Before proceeding with autopsy, fourteen swabs were taken around the victim’s skin area near the dog’s bite marks in order to obtain dog saliva and DNA. The α-amylase test for the salivary enzyme presence could not be performed in victim’s wounds because salivary amylase is lacking or at very low abundance in mammalian carnivores such as cats and dogs [9, 10]. Regardless, the authors proceeded with swabbing the clearest bite-marks. Cardiac peripheral blood was taken to obtain the victim’s DNA reference sample. The biting victim’s clothes were packed and stored to be analyzed in a forensic genetic laboratory.

**Fig. 3:**
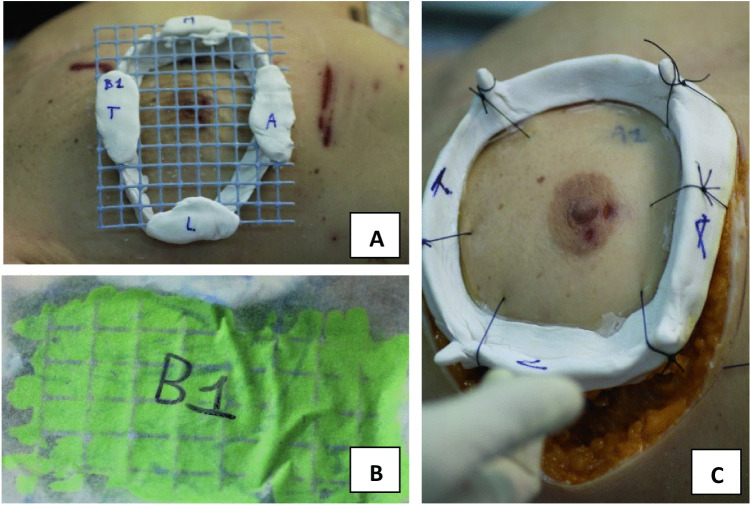
Silicone cast making and anatomic sample collection. **A** The methacrylate ring was applied around the bitten right Montgomery’s areola. **B** High-viscosity silicone was added to completely fill the containment ring, remained in place until polymerization was complete, and the bite silicone marks were obtained. **C** After the silicone was removed, the anatomical area was sutured to the methacrylate ring at several points, and then cut to the level of the muscular plane to stabilize the skin to the ring for further comparisons

Subsequently, the “right Montgomery’s areola,” the “upper left hypochondriac area,” and the “left mid-tibial area” cutaneous bite injuries were selected for further specific analysis. The three anatomic areas were respectively numbered as AA1, AA2, and AA3 (Table 1), and their resulting bite imprints (B1, B2, and B3) were produced. A self-curing methacrylic resin ring was used to surround and border the damaged area. A high-viscosity addition silicone was added to completely fill the containment ring and remain in place until the polymerization was complete, and the bite silicone mark was obtained. After the silicone mark was removed (Fig. 3), it was sent to an odontological laboratory for a casting class IV hard plaster impression. Each anatomical area, AA1, AA2, and AA3, was again bordered with self-curing methacrylic resin, and the obtained ring was adhered to the skin with cyanoacrylate-based compound. Afterwards, a clean incision was made with a 22-blade scalpel from the skin to the muscle following the methacrylic resin ring border. The resulting muscle-cutaneous tissue containing the bite lesion was stabilized to the methacrylic resin ring by means of single, circular sutures for further comparisons. This activity was carried out in accordance with the guidelines of the American Board of Forensic Odontology (ABFO) [11], always taking care to avoid distortion of the tissue in order to photographically preserve the color and depth of the underlying bruises. Furthermore, cutaneous muscle samples were fixed in a solution of 5 mL 40% formaldehyde, 5 mL 99.8% glacial acetic acid, and 90 mL 7% ethanol. The samples were then stored for a period of 1 week after which they were removed from the formaldehyde bath and monitored for changes in dimension and stability, as well as their adherence or loss to the rings. The examined impressions of the dental arches on the skin were subjected to metric evaluations for subsequent comparative purposes.

**Table 1:** Skin lesion and corresponding Dog dental cast matches. Dog_2 was directly implicated in AA1 and AA2 injurie production present on the victim’s body. Its dental cast matched with these two anatomical lesions. Dog_1 was directly implicated in AA3 bite injury production. Its dental cast matched that anatomical lesion

Anatomical region	Excised anatomical region	Silicone bite imprints	Matched dental cast	Dog
Right Montgomery’s areola	AA1	B1	B2mg	2
Upper left hypochondriac area	AA2	B2	B2mg	2
Left mid-tibial area	AA3	B3	B1mg	1

### Investigation of dogs’ bite marks analysis

**Fig. 4:**
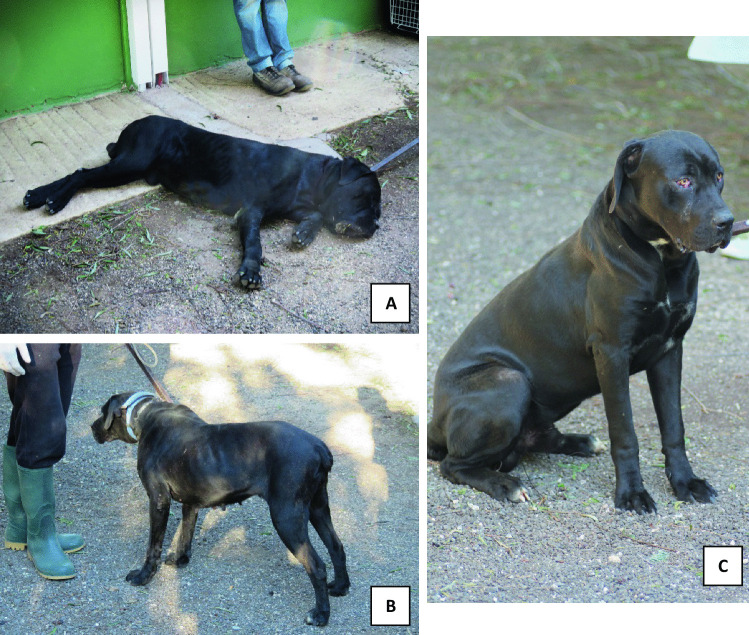
Three of the six Cane Corso dogs subjected to judicial seizure before forensics veterinaries, odontological and genetics analysis were done at kennel. **A** Dog_1 was reported to be the alpha dog of his pack, a mixed saliva-blood substance was found in his mouth during the first on-site examination. **B** Dog_2 was the mother of the two puppies: Dog_3 and Dog_4. During the kennel investigations, a piece of fabric matching the victim’s shirt was found in her excrement. **C** Dog_3 was one of the juvenile dogs

The six Cane Corso dogs (Fig. 4) were taken to the kennel and subjected to judicial seizure. An initial analysis of the two dog groups showed that Dog_3 and Dog_4 were Dog_1 and Dog_2’s puppies, in adolescent stage (6–18 months). Whereas, the other two dogs were not related to the first four. After veterinary microchip recognitions and dog anesthetization, the oral mucosa cells were swabbed in order to obtain each dog’s reference sample, and the upper and lower dental impression were taken by modified steel dental tray. Before each dog was awakened, the dental formula was calculated (Table 2). Three dogs of the first owner (Dog_2, Dog_3, and Dog_4) had missing teeth. In addition, three of the six dogs (Dog_2, Dog_4, and Dog_6) exhibited a third-class malocclusion; this alteration is known as dysgnathia, where the lower jaw appears to be advanced compared to the upper jaw.

**Table 2:** The dental formula of six Cane Corso dogs and related dental cast code. Dog_2, Dog_3, and Dog_4 presented lower jaw incisive anomalies (*). Dog_2, Dog_4, and Dog_6 were diagnosed with a class III dysgnathia. Dysgnathia was not detected (N.D.) in Dog_1, Dog_3, and Dog_5. Dental formula: I, incisors; C, canines; P, premolars; M, molars. Fraction slash (/) was used to divide left arch teeth from right arch teeth of each jaws

Dog	Dysgnathia	Upper dental formula	Lower dental formula	Dental cast code
1	N.D	I 3/3, C 1/1, P 4/4, M 2	I 3/3, C 1/1, P 4/4, M 2	B1mg
2	III class	I 3/3, C 1/1, P 4/4, M 2	I 2*/3, C 1/1, P 4/4, M 2	B2mg
3	N.D	I 3/3, C 1/1, P 4/4, M 2	I 3/2*, C 1/1, P 4/4, M 2	B3mg
4	III class	I 3/3, C 1/1, P 4/4, M 2	I 3/2*, C 1/1, P 4/4, M 2	B4mg
5	N.D	I 3/3, C 1/1, P 4/4, M 2	I 3/3, C 1/1, P 4/4, M 2	B5mg
6	III class	I 3/3, C 1/1, P 4/4, M 2	I 3/3, C 1/1, P 4/4, M 2	B6mg

Six sodium alginate canine dental impressions from Dog_1 to Dog_6 were obtained and numbered as follows: B1mg, B2mg, B3mg, B4mg, B5mg, and B6mg. A detailed photograph and analysis of each dog’s jaw was taken following ABFO recommendations [11]. Inter-canine distances and canine heights on each cast were also recorded using a digital caliper (Table 3). A piece of rose and green striped shirt was collected from Dog_2 excrement and preserved for further comparisons. Each dog dental cast was compared to the victim’s lesions by mechanical projection and by using DentalPrint^©^ software [12].

**Table 3:** Dog dental anatomy measurements on dental casts. Upper inter-canine distance (U.I.C.D) is the distance measured in mm between canine in the upper cast. Lower inter-canine distance (L.I.C.D) is the distance measured in mm between canine in the lower cast. Upper canine height (U.C.H.) is the upper cast canine’s height expressed in mm. Lower canine height (L.C.H.) is the lower cast canine’s height expressed in mm

Dog	Dental cast	U.I.C.D	L.I.C.D	U.C.H	L.C.H
1	B1mg	55 mm	45 mm	19 mm	13 mm
2	B2mg	52 mm	50 mm	20 mm	18 mm
3	B3mg	48 mm	44 mm	18 mm	14 mm
4	B4mg	55 mm	41 mm	22 mm	15 mm
5	B5mg	53 mm	42 mm	18 mm	15 mm
6	B6mg	49 mm	43 mm	16 mm	14,5 mm

### Dog DNA genotyping

The bitten victim’s clothes (e.g., blue jeans) together with dogs’ reference DNA were used to obtain the dogs’ DNA genetic profiles. DNA extraction was carried out using the QIAamp^®^ DNA Mini Kit [13]. A preliminary amplification was performed on the extracts with universal primers for the canine mitochondrial cytochrome b gene [14–17]. This amplification provided information on the animal species that was eventually identified through sampling. The Dog DNA STR amplification was carried out using a ThermoFisher™ Canine STR panel 1.1 kit [18], composed of 18 autosomal loci and the amylogenic locus for sex determination. These regions are recommended by the International Society for Animal Genetics (ISAG) [19].

### Human DNA genotyping

The victim’s clothes collected during the autopsy were initially observed by forensic lights. For presumptive trace detection of bloodstains [20], the Roche^®^ tetramethylbenzidine (TMB) Combur^3^Test^®^ was used. The samples that tested positive to the Combur^3^Test^®^ reaction were subjected to the human blood detection by Bluestar^®^ OBTI Immunochromatographic test [21]. All the samples that gave a positive result in the Bluestar^®^ OBTI test, the blood collected from the Dog_1’s mouth and the victim’s control cardiac blood (taken during the autopsy), were used for the human DNA extraction using the QIAamp^®^ DNA Mini Kit [13]. Human DNA quantification was conducted by the Quantifiler^®^ Human kit [22]. Human STR amplification were conducted on a ThermoFisher GeneAmp^®^ PCR System 9700 amplifier, and STR amplification was obtained using the GlobalFiler™ PCR Amplification Kit. The amplified products were separated into capillary electrophoresis with the 3500 Series Genetic Analyzer sequencer by Applied Biosystems™. Alleles were assigned by GeneMapper ID-X Software v1.1.2. C

## Results and discussion

### Autopsy results

During the analysis of the victim’s head, the only interesting element that appeared was a subgaleal ecchymosis indicating a contusion compatible with the scenario. Most of the victim’s body had multiple superficial and deep tissue lacerations. Specifically, the right brachial artery was found slashed. The fatal blood loss was found in the right limb correspondence, where the dogs had bitten and slashed the victim’s tissues massively and repeatedly. In general, the upper limbs were repeatedly bitten, a condition likely resulting from the victim’s defensive posture. The subsequent thoraco-abdominal section of the cadaver did not reveal anything of significance from a traumatic point of view. Anatomopathological studies of the victim’s organs and tissue fragments revealed bilateral calcific coronary atherosclerosis. The left coronary artery showed 70–75% stenosis, a marked congestive phenomenon, and small hemorrhagic stasis in the epicardial area. Toxicological analyses carried out on the victim’s blood and urine did not reveal the presence of psychotropic and narcotic substances. The victim’s death resulted from hemorrhagic and traumatic shock caused by the deep dog bite wounds at the brachial level.

### Dogs’ bite marks results

**Fig. 5:**
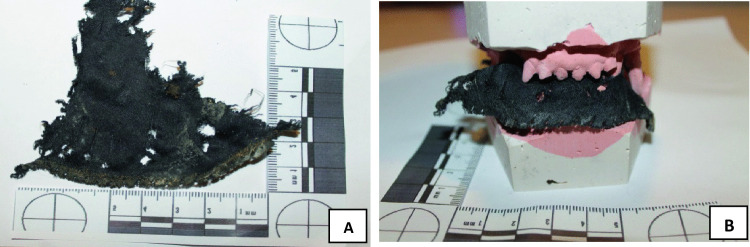
Mechanical comparison between clothes fragments and dog dental chalk. **A** Victim’s sweater fragment, found and collected on death scene, showed various holes attributable to a dog bite. **B** The mechanical comparison between the sweater fragment (**A**) and Dog_1’s dental chalk provided a full compatibility. This comparison was confirmed also measuring the upper and lower inter-canine distance (55 mm and 45 mm, respectively)

The dog sodium alginate dental casts were mechanically projected into the excised anatomical areas or bitten clothes. A comparison between each cast and the silicone bite imprints was attempted in order to identify which dog had the greatest responsibility in the victim’s death. A morphologically positive concordance between the AA1, a fragment of the victim’s black sweater, and the B1mg was detected (Fig. 5). A second analysis using Adobe Photoshop^®^ [23] and DentalPrint^©^ software [12] was conducted in order to confirm a positive match between B1 and B1 mg, confirming the presence of Dog_1 in the attack that took the victim’s life. The presence of Dog_1 was also confirmed by projecting B1mg onto a fragment of the victim’s clothing (Table 4), and B1mg and B1 onto AA3. The calculated inter-canine distance from B1mg also confirmed the same Dog_1’s bite mark in AA3 (Table 1).

**Table 4:** Clothes fragments and corresponding dog dental cast matches. Dog_2 was directly implicated in C1 and C2 bits on the victim’s clothes. Its dental cast matched these two samples. Dog_1 was directly implicated in C3 bits on victim’s clothes. Its dental cast matched this sample

Victim’s clothes	Sample code	Matched dental cast	Dog
Black sweater fragment	C1	B2mg	2
Rose/green stripes shirt fragment	C2	B2mg	2
Inner jacket shoulder fragment	C3	B1mg	1

**Fig. 6:**
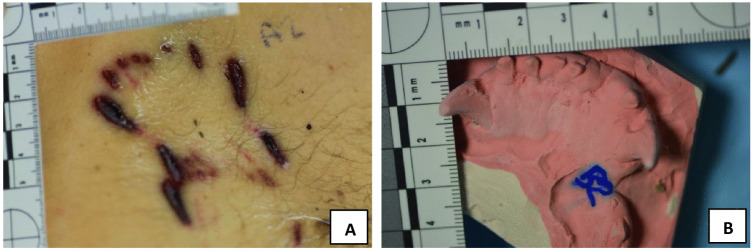
The comparison between upper left hypochondriac area injury (**A**) and the B2mg under jaw cast (**B**). These findings were confirmed by a visible missing tooth in the skin injury (**A**), in the cast (**B**), and also by measuring the lower inter-canine distances (50 mm)

Furthermore, AA2 was examined, and a morphologically positive concordance with B2mg was detected by a missing lower incisive in Dog_2’s bite mark. The same concordance was obtained by matching B2 with B2mg using Adobe Photoshop^®^ [16] and DentalPrint^©^ software [17], confirming the presence of Dog_2 during the attack (Table 1). Due to the pronounced dysgnathia, the Dog_2’s bite was easier identifiable on victim’s skin (Fig. 6) and victim’s clothing (Table 4). The presence of Dog_2 on the death scene was also confirmed by the shirt fragment found in the excrement recovered 48 h after the animal’s seizure at the judicial kennel. The fragment resulted to be part of the victim’s shirt worn during the assault.

The dog dental arche analyses on the victim’s skin were compared by mechanical projections showing that the lesions AA1/B1 and AA2/B2 were fully compatible with the plaster model B2mg that belonged to Dog_2. Dog_2’s bite was easily identifiable because of the pronounced dysgnathia and prevailing indentations of the lower jaw teeth. Dog_2’s bite presented another peculiarity that led to rapid identification in AA2, these being a missing incisor in the inferior dental arch.

### DNA genotyping results

Dog DNA reference results confirmed that Dog_1 and Dog_2 were parents of Dog_3 and Dog_4, and excluding any familial relationship to Dog_5 and Dog_6. Moreover, Dog_2’s DNA was found in two pieces of victim’s trousers, connecting that dog on to the death scene. The blood found in Dog_1’s mouth directly belonged to victim, connecting Dog_1 to the victim’s injuries. Additionally, DNA comparisons between the victim’s reference DNA and the DNA extracted from the blooded clothes demonstrated that they were worn by the victim during the attack. No evidence of Dog_5 and Dog_6’s dental arches were found on the victim’s skin and clothes. Additionally, no salivary DNA of these dogs was found on all examined samples, demonstrating their absence during the attack. For this reason, they were considered fully innocent and were immediately released from the kennel. Likewise, Dog_3 and Dog_4 were released from the kennel because the victim’s injuries did not present positive matches with their dental imprints. Instead, their parents’ massive (Dog_1 and Dog_2) interaction were confirmed by various factors. Dog_1 had positive matches between its dental chalk and a victim’s clothes fragment (C3) (Fig. 5) and a skin injury (AA3). Also, the victim’s blood was found in Dog_1’s upper jaw. Dog_2’s informative jaws anomalies, caused by dysgnathia and a missing lower tooth, easily connect the dog to the attack, its dental chalk matched with two of the victim’s clothes fragments (C1 and C2). Additionally, a piece of rose and green striped shirt was recollected from Dog_2’s excrement.

## Conclusion

To conclude, in almost 50% of dog bite cases described in literature [24–26], as in our case, the attacks took place near or inside the dog owner’s property. The dog pack familiarity with the agriculture plot was confirmed by the presence of black dog hair tufts on the rusty mesh wire hole; this suggests that the hole was frequently used by dogs to pass from one property to another. None of the dogs had ever shown aggression toward humans. They were also in daily contact with their owner’s four-year-old daughter. To note, Dog_3 and Dog_4 were in the late or second stage of socialization, also called the juvenile stage [27]. The juvenile period begins between the fourteen and sixteen weeks of age and ends with the onset of puberty, which in larger breeds such as the Cane Corso has a longer than usual period, up to 1 year of age [28, 29]. During this time, adolescent dogs begin to feel more comfortable interacting with people and other animals [27]. Curiosity may have led them to approach the victim, who was unfamiliar with the agricultural plot and the dogs. The adult dogs, probably reacting with an aggressive attitude, may have attacked the victim to protect the weaker juveniles. The protection may have resulted from the fact that the dogs Dog_3 and Dog_4, in the phase of sexual immaturity, did not yet have any hierarchies in the group and were considered protected by Dog_1 and Dog_2. Unfortunately, no witnesses were able to indicate whether the dogs were sending alarm signals to the victim and he ignored them, or whether the victim was acting aggressively toward the dogs who, feeling threatened, attacked him. In conclusion, the reported event has all the elements of a fatal dog pack attack as a result of a probably territorial and pack defense against intruders.

## Key points

- To date, no forensic cases of death from dog pack attack have been solved by dog dysgnathia conditions.
- Crime scene analysis and autopsy cannot assign discriminative dog bite marks, therefore odontological, and genetic considerations should be always taken in account.
- A forensic multidisciplinary approach was necessary to solve the case.
- The dogs’ STRs and dental anomalies were fundamental to the recognition of those that induced victim death.

## References

[1] Salem NH, Belhadj M, Aissaoui A, Mesrati MA, Chadly A. Multidisciplinary approach to fatal dog attacks: a forensic case study. J Forensic Leg Med. 2013;20:763–6. 10.1016/j.jflm.2013.04.015. 23910877 10.1016/j.jflm.2013.04.015

[2] Mora E, Fonseca GM, Navarro P, Castaño A, Lucena J. Fatal dog attacks in Spain under a breed-specific legislation: a ten-year retrospective study. J Vet Behav. 2018;25:76–84. 10.1016/j.jveb.2018.03.011.

[3] Avis SP. Dog pack attack: hunting humans. Am J Forensic Med Pathol. 1999;20:243–6. 10.1097/00000433-199909000-00005. 10507791 10.1097/00000433-199909000-00005

[4] Borchelt PL, Lockwood R, Beck AM, Voith VL. Attacks by packs of dogs involving predation on human beings. Public Health Rep. 1983;98:57–66. 6828639 PMC1424390

[5] De Munnynck K, Van de Voorde W. Forensic approach of fatal dog attacks: a case report and literature review. Int J Legal Med. 2002;116:295–300. 10.1007/s00414-002-0332-9. 12376842 10.1007/s00414-002-0332-9

[6] Fonseca GM, Palacios R. An unusual case of predation: dog pack or cougar attack? J Forensic Sci. 2013;58:224–7. 10.1111/j.1556-4029.2012.02281.x. 22971181 10.1111/j.1556-4029.2012.02281.x

[7] Kneafsey B, Condon KC. Severe dog-bite injuries, introducing the concept of pack attack: a literature review and seven case reports. Injury. 1995;26:37–41. 10.1016/0020-1383(95)90550-H. 7868208 10.1016/0020-1383(95)90550-h

[8] Sarenbo S, Svensson PA. Bitten or struck by dog: a rising number of fatalities in Europe, 1995–2016. Forensic Sci Int. 2021;318: 110592. 10.1016/j.forsciint.2020.110592. 33246867 10.1016/j.forsciint.2020.110592

[9] Pasha S, Inui T, Chapple I, Harris S, Holcombe L, Grant MM. The saliva proteome of dogs: variations within and between breeds and between species. Proteomics. 2018;18:1–7. 10.1002/pmic.201700293. 10.1002/pmic.201700293 PMC5969230 29327448

[10] Boehlke C, Zierau O, Hannig C. Salivary amylase - the enzyme of unspecialized euryphagous animals. Arch Oral Biol. 2015;60:1162–76. 10.1016/j.archoralbio.2015.05.008. 26043446 10.1016/j.archoralbio.2015.05.008

[11] David TJ, Golden GS, Loomis PW, Freeman A, Berman G. American Board of Forensic Odontology Diplomates Reference Manual. 2012;67–75:107–18.

[12] Valenzuela-Garach JC. Aurora; Martín de llas Heras, Stella; Torres-Cantero, Manual De Usuario De DentalPrint^©^ software. 2001.

[13] QIAGEN, QIAamp DNA Mini and Blood Mini Handbook, Qiagen. 2016;1–72. http://www.qiagen.com/knowledge-and-support/resource-center/resource-download.aspx?id=67893a91-946f-49b5-8033-394fa5d752ea&lang=en.

[14] Rahman MM, Ali ME, Hamid SBA, Mustafa S, Hashim U, Hanapi UK. Polymerase chain reaction assay targeting cytochrome b gene for the detection of dog meat adulteration in meatball formulation. Meat Sci. 2014;97:404–9. 10.1016/j.meatsci.2014.03.011. 24769096 10.1016/j.meatsci.2014.03.011

[15] Steinlechner M. Species identification by means of the cytochrome b gene. Int J Legal Med. 2000;23–28. 10.1007/s004140000134 11197623

[16] Awad A, Khalil SR, Abd-Elhakim YM. Molecular phylogeny of some avian species using Cytochrome b gene sequence analysis. Iran J Vet Res. 2015;16:218–22. 27175180 PMC4827690

[17] Bradley RD, Baker RJ. A test of the genetic species concept: Cytochrome-b sequences and mammals. J Mammal. 2001;82:960–73. 10.1644/1545-1542(2001)082%3c0960:ATOTGS%3e2.0.CO;2.

[18] Thermo Fisher Scientific. Thermo Scientific Thermo Scientific Canine Genotypes Panel. 2012;1.1(0):1–39.

[19] Budowle B, Garofano P, Hellman A, Ketchum M, Kanthaswamy S, Parson W, Van Haeringen W, Fain S, Broad T. Recommendations for animal DNA forensic and identity testing. Int J Legal Med. 2005;119:295–302. 10.1007/s00414-005-0545-9. 15834735 10.1007/s00414-005-0545-9

[20] De Vittori E, Barni F, Lewis SW, Antonini G, Rapone C, Berti A. Forensic application of a rapid one-step tetramethylbenzidine-based test for the presumptive trace detection of bloodstains at the crime scene and in the laboratory. Forensic Chem. 2016;2:63–74. 10.1016/j.forc.2016.10.002.

[21] Obti B. Test I. Bluestar^®^ obti. 2021;98000.

[22] Biosystems A. Quantifiler ^TM^ HP and Trio DNA Quantification Kits. 2017;4485354;116. https://assets.thermofisher.com/TFS-Assets/LSG/manuals/4485354.pdf.

[23] Chintala L, Manjula M, Goyal S, Chaitanya V, Hussain MKA, Chaitanya YC. Human bite marks - a computer-based analysis using adobe photoshop. J Indian Acad Oral Med Radiol. 2018;30:58–63. 10.4103/jiaomr.jiaomr-87-17.

[24] Beck AM, Loring H, Lockwood R. The ecology of dog bite injury in St. Louis, Missouri. Pub Hlth Rep. 1975;90:262–7. PMC1435667 807942

[25] Winkler WG. Human deaths induced by dog bites, United States, 1974–75. Public Health Rep. 1977;92:425–9. 910019 PMC1432043

[26] Pinckney LE, Kennedy LA. Traumatic deaths from dog attacks in the United States. Pediatrics. 1982;69:193–6. 10.1542/peds.69.2.193. 7058093

[27] Dietz L, Arnold AMK, Goerlich-Jansson VC, Vinke CM. The importance of early life experiences for the development of behavioural disorders in domestic dogs. Behaviour. 2018;155:83–114. 10.1163/1568539X-00003486.

[28] Battaglia CL. Periods of early development and the effects of stimulation and social experiences in the canine. J Vet Behav Clin Appl Res. 2009;4:203–10. 10.1016/j.jveb.2009.03.003.

[29] Howell T, King T, Bennett P. Puppy parties and beyond: the role of early age socialization practices on adult dog behavior. Vet Med Res Rep. 2015;143. 10.2147/vmrr.s62081. 10.2147/VMRR.S62081 PMC6067676 30101101

